# Understanding Violent Radicalization and Conspiracy Belief in Dutch Youth Aged 16–25: A Latent Profile Analysis

**DOI:** 10.1007/s10964-025-02250-4

**Published:** 2025-09-17

**Authors:** Jessica I. den Elzen, Jessica J. Asscher, Kyle M. Lang, Hanne M. Duindam

**Affiliations:** 1 Department of Clinical Child and Family Studies, Utrecht University, Utrecht, the Netherlands; 2 Department of Methodology and Statistics, Utrecht University, Utrecht, the Netherlands

**Keywords:** Violent radicalization, Conspiracy belief, Emerging adulthood, Middle to late adolescence, Latent profile analysis

## Abstract

Violent radicalization linked to conspiracy belief has gained increasing attention over the last few years, yet little is known about these dynamics in youth. In this study, a latent profile analysis was conducted to better understand violent radicalization in the context of conspiracy belief among youth aged 16–25. Participants were 2297 Dutch adolescents and emerging adults (*Mage* = 19.55, *SDage* = 2.75; 63.3% female). Six profiles were identified, varying in levels of violent radicalization and conspiracy belief. While the largest profile indicated low levels of both, two smaller profiles showed heightened violent radicalization, with differing conspiracy belief. Heightened radicalization profiles were generally younger, male, and more politically extreme, whereas vocational education was more prevalent in high conspiracy profiles. Overall, the findings highlight different manifestations of violent radicalization and conspiracy belief in middle to late adolescence and emerging adulthood and the importance of considering individual and broader contextual conditions.

## Introduction

Violent radicalization linked to conspiracy belief has gained increasing attention from policymakers, governments and researchers (Basit, 2021; Kruglova, 2023). High-profile incidents, such as the vandalism of 5G masts in Europe (Basit, 2021) and the 2021 Capitol Hill riots (Armaly et al., 2022) have been linked to misinformation and conspiracy theories. While most conspiracy believers do not seem to turn to violence (Moskalenko & McCauley, 2021), the potential for a small subset to do so remains a pressing concern. Violent radicalization based on conspiracy belief can have large-scale effects. In addition to direct material consequences, this type of violent radicalization also poses a broader threat to democracy by undermining trust in institutions (Jahnke et al., 2022). Prior research on youth on this topic has been limited, despite young people’s vulnerability to both exposure to conspiracy belief (Enders et al., 2021) and online recruitment by violent radical groups (Neumann & Rogers, 2007). Therefore, the purpose of this study is to better understand how violent radicalization in the context of conspiracy belief manifests in middle to late adolescence and emerging adulthood. More specifically, the first aim is to explore which profiles of youth (aged 16–25) can be identified based on violent radicalization (attitudes, intentions, and behavior) and conspiracy belief (specific and general). The second aim is to further explore intergroup differences on characteristics related to violent radicalization and conspiracy belief, specifically: gender, age, education type, migration background, financial strain, extreme political orientation, and psychological distress.

### Adolescence and Emerging Adulthood

Disagreeing with dominant political ideas or questioning the status quo are healthy parts of democratic societies, particularly among youth, for whom civic engagement and critical thinking are key aspects of development (Law & Atkinson, 2021). However, concerns arise when this comes to involve the justification or use of violence to achieve political or societal aims. Youth between the ages of 16 and 25 are in the developmental stages of middle to late adolescence (Hardin et al., 2017) and emerging adulthood (Arnett, 2000). For brevity, this age group will throughout the text also be referred to as ‘youth’ or ‘adolescents and emerging adults’. Youth between the ages of 16 and 25 are in a transitional life stage. They are facing more adult challenges than younger individuals, but often without the cognitive maturity or coping tools that older age groups possess (Wilens & Rosenbaum, 2013). Both conspiracy theories and violent radical groups are especially attractive to this age group as they offer a sense of significance (Kruglanski et al., 2014), a simplistic narrative of how the world works (Marchlewska et al., 2018), social ties (Dhami & Murray, 2016), excitement (Van Prooijen et al., 2022), and a sense of adventure (Schumpe et al., 2020), all of which tie into this group’s developmental period, characterized by identity development, feelings of uncertainty, and a need to belong (Oppetit et al., 2019). Violent radicalization may result from adverse processes throughout one’s development (i.e., social-developmental model, Beelmann, 2020), with ontogenetic factors emerging from early childhood through early adulthood influencing the likelihood that more proximal radicalization processes are triggered during adolescence and early adulthood. This situates the age group of this study within these critical developmental windows, underscoring the importance of studying violent radicalization in this population.

### Violent Radicalization and Conspiracy Belief

Violent radicalization refers to the process of individuals increasingly justifying violence as a way to achieve political or societal change (Doosje et al., 2016; Orsini, 2023). This includes violent radical attitudes (i.e., sympathy, legitimization, agreement, or justification of violence), violent radical intentions (i.e., intent or willingness to engage in violence), and violent radical behavior (i.e., committing or having committed violence, including terrorism). All three components are distinct manifestations of violent radicalization. These components may co-occur but not necessarily, as argued in the two-pyramids model (McCauley & Moskalenko, 2017). This model distinguishes between radicalization of opinion (cognitive radicalization) and radicalization of action (behavioral radicalization). According to this model, cognitive radicalization does not always lead to behavioral radicalization, which makes it important to examine them all as separate yet potentially interconnected processes, instead of assuming one inevitably leads to the other. Moreover, recent literature has also highlighted that the strength of risk and protective factors can vary across these different manifestations of violent radicalization (Wolfowicz et al., 2021). For example, they found that being male and unemployed was more predictive of violent radical behavior than of attitudes or intentions, while factors such as anger and perceived injustice had stronger associations with violent radical intentions than with attitudes. Together, these findings highlight the importance of including violent radical attitudes, intentions, and behavior to more accurately capture all different manifestations of violent radicalization.

Conspiracy theories are defined as the conviction, regardless of accuracy, that a group or individual secretly influences or has influenced large-scale, politically or socially significant events, typically with harmful consequences (Imhoff & Lamberty, 2020). In this study, a distinction is also made between general conspiracy belief, which refers to a more generalized worldview of the presence of conspiracy theories, and specific conspiracy belief, which refers to belief in the veracity of a specific conspiracy theory. Research suggests that these concepts may differ in several ways (Imhoff, Bertlich, et al., 2022). General conspiracy belief seems to be more normally distributed, content-independent, and stable over time, whereas specific conspiracy belief is more skewed with stronger beliefs being limited to few individuals, and more content-dependent and susceptible to change. While questioning dominant narratives can foster healthy skepticism, conspiracy beliefs also often involve distrust of credible evidence and institutions and have been linked to negative outcomes such as political disengagement (Jolley & Douglas, 2014), vaccine hesitancy (Earnshaw et al., 2020), lower intention to reduce climate footprint (Jolley & Douglas, 2014), and, in some cases, violent radicalization (e.g., Vegetti & Littvay, 2022).

### Theoretical Framework

The 3N model of radicalization (Kruglanski et al., 2019) has incorporated the role of conspiracy belief into their model and explained how conspiracy belief contributes to violent radicalization through needs, narratives, and networks (Kruglanski et al., 2022). *Needs* refers to a need for significance, so the need for meaning, purpose, and importance in life. This need can be provoked by a loss of significance, such as humiliation of an individual or social group, or an opportunity to (re)gain significance, such as through identifying, and sometimes fighting, an alleged enemy. Conspiracy beliefs can amplify this need by framing events as grievances and identifying scapegoats. These beliefs shape *narratives* that describe the grievances and scapegoats, offering, implicitly or explicitly, a way to (re)gain significance, sometimes through violence. Conspiracy theories often serve as such narratives and are frequently used in the rhetoric and calls to action of violent radical groups (Rousis et al., 2022). Finally, *networks* reinforce violent radicalization by validating these narratives and encouraging or rewarding (violent) action in response to these narratives. Conspiracy communities are a particularly tight-knit network as they amplify shared beliefs and marginalize or discredit individuals who criticize these beliefs. In this way, conspiracy belief can play a role in all three components of the 3N model, contributing to violent radicalization.

### Previous Research on Violent Radicalization and Conspiracy Belief

Research has previously shown an overall association between conspiracy belief and violent radicalization in adults (Belton et al., 2025). Specifically, general conspiracy belief has been associated not only with justifications for general political violence (Vegetti & Littvay, 2022) but also with specific violent intentions tied to conspiracy theories, such as arson of 5G towers (Jolley & Paterson, 2020). This was further supported by an experimental study that found people who were asked to imagine living in a society high in conspiracies reported stronger violent radical intentions than those who imagined living in a society low in conspiracies (Imhoff et al., 2021).

Research on specific conspiracy belief further supports the link between conspiracy belief and violent radicalization in adults. For example, belief in various conspiracy theories has been associated with positive attitudes towards and intentions to engage in violent anti-government protests (Šrol et al., 2022). Furthermore, belief in QAnon conspiracy theories was associated with violent radical attitudes and intentions, both general and in the context of the Capitol Hill riots in 2021 (Moskalenko et al., 2023) and QAnon supporters were more likely to endorse violent protests and interpersonal political violence compared to non-supporters (Sandlin & Garland, 2024). Similarly, belief in the “Great Replacement” conspiracy theory, which claims that mass immigration is deliberately replacing White Westerners, predicted both violent radical intentions and attitudes, including support for the violent persecution of Muslims (Obaidi et al., 2022). Finally, belief in COVID-19 conspiracy theories was not only associated with violent radical attitudes and intentions specific to COVID-19 (Jolley & Paterson, 2020; Šrol et al., 2022) but also with more general violent radical attitudes and intentions (Levinsson et al., 2021; Šrol et al., 2022). These findings suggest that belief in specific conspiracy theories may not only justify violence toward the alleged actors and implicated groups but also relate to more general violent radical attitudes and intentions.

While the studies above have examined violent radical attitudes and intentions, few have explored violent radical behavior, despite this being another important component of violent radicalization (McCauley & Moskalenko, 2017). Furthermore, most research has focused on adult populations, even though youth may be particularly susceptible to conspiracy belief (Enders et al., 2021) and violent radicalization (Neumann & Rogers, 2007). Finally, prior studies have largely relied on variable-centered approaches, which examine associations between constructs but may overlook meaningful patterns across individuals. Yet, as research has shown, not all individuals who believe in conspiracy theories seem to violently radicalize (Moskalenko & McCauley, 2021) and those who do may follow different trajectories. Person-centered methods have shown promise in this field in capturing these complexities (e.g., Duindam et al., 2025; Miconi et al., 2024), as this method allows for the identification of profiles of individuals which can help identify patterns of resilience and vulnerability in violent radicalization and conspiracy belief.

### The Context of Conspiracy Belief and Violent Radicalization

It is important to consider that violent radicalization and conspiracy belief arise within a context. Socio-ecological models (Bronfenbrenner, 2005), have been proposed as useful frameworks in this domain (Miconi et al., 2024), framing violent radicalization as stemming from the interactions between the micro- meso-, and macro-level, ranging from personal experiences to broader systemic conditions. Others have also argued to move away from a criminal justice framework of violent radicalization to a framework of public health, focusing more on structural and systematic factors (Eisenman & Flavahan, 2017). The 3N model of radicalization also highlights how needs can be triggered or intensified by broader contextual stressors, such as discrimination, financial hardship, or political disillusionment (Kruglanski et al., 2022). In this context, gender, age, education type, migration background, financial strain, political orientation, and psychological distress are not just demographic and individual characteristics, they are indicative of people’s broader context and experiences in society, which could create conditions that trigger the need for significance.

Prior research supports this contextualized view, showing that violent radicalization and conspiracy belief tend to co-occur with these characteristics. Violent radicalization has been associated with male gender (Levinsson et al., 2021), younger age (Pfundmair et al., 2021), increased financial strain (Thijs et al., 2024), more extreme political orientation (Wagner-Egger et al., 2022), and higher psychological distress (Adam-Troian & Bélanger, 2024). Findings regarding education and violent radicalization are mixed, with some studies linking it to lower education levels (Šrol et al., 2022), and others to higher education levels (Levinsson et al., 2021). Results are also inconsistent for migration background, with some studies finding stronger violent radicalization among those with a migration background (Thijssen et al., 2023), whereas others report the opposite (Levinsson et al., 2021). Similarly, conspiracy belief has been associated with lower education (Van Prooijen, 2017) increased financial strain (Walter & Drochon, 2022), extreme political orientation (Imhoff, Zimmer, et al., 2022), and higher psychological distress (Jensen & Kane, 2024). Findings on gender, age, and migration background are mixed, with some studies finding stronger conspiracy belief in men (Walter & Drochon, 2022), younger people (Galliford & Furnham, 2017), and those with a migration background (Van Prooijen et al., 2018), whereas others find this in women (Moskalenko et al., 2023), older people (Walter & Drochon, 2022), and those without a migration background (Levinsson et al., 2022). Research further suggests that these conditions can interact. For instance, the relationship between conspiracy belief and violent radicalization appears stronger among men (Moskalenko et al., 2023) and among those reporting higher psychological distress (Levinsson et al., 2021), and depressive symptoms (Baum et al., 2024). By examining profile differences across these factors, this study aims to better understand differing social contexts and unmet needs among adolescents and emerging adults, and how these factors might shape violent radicalization and conspiracy belief during this critical developmental stage.

Importantly, these factors must be considered within the broader Dutch socio-political context during the period of data collection (May 2023-November 2024). Based on national surveys, it was concluded that Dutch youth, aged 18–25, report lower personal wellbeing than adults, specifically in terms of trust in institutions and financial future, with only about a third of youth reporting high wellbeing on these aspects (Centraal Bureau voor de Statistiek, 2024). Dutch youth were also more likely to engage in political protests than adults, with 44% of youth between 18 and 25 indicating they had engaged in a form of political protest in the last five years (Centraal Bureau voor de Statistiek, 2024). Generally, polls indicate that trust in politicians has declined over time (Centraal Bureau voor de Statistiek, 2025). Some notable political and social events at the time of data collection include the fall of the Rutte IV government (NOS, 2023), with a significant shift toward more populist right-wing parties in the following elections (Otjes & De Jonge, 2024), and an increase in protests, such as climate activism by Extinction Rebellion (NOS, 2024a), growing pro-Palestinian protest activity (NOS, 2024b), and farmers’ protests related to nitrogen emission regulations (Finger et al., 2024).

## Current Study

Prior research on violent radicalization and conspiracy belief has largely focused on adults, relied on variable-centered approaches, and rarely assessed behavior. The present study addresses these gaps by focusing on adolescents and emerging adults, aged 16–25, a population particularly vulnerable to conspiracy theories and violent radical groups due to developmental needs for belonging, excitement, and identity exploration. Furthermore, by using a person-centered approach, the study captures interpersonal heterogeneity and identifies distinct profiles based on patterns of conspiracy belief and violent radicalization. This approach offers a better understanding and may inform the development of more targeted and effective interventions. Finally, it includes a self-report measure of violent radical behavior, in addition to measures of violent radical attitudes and intentions, to provide a more comprehensive overview of the different components of violent radicalization. This study used a large-scale survey of Dutch adolescents and emerging adults (ages 16–25), recruited through schools, social media platforms, and political events, to achieve a diverse sample that represents as many Dutch youth as possible. Overall, it was expected that most youth would report low levels of both violent radicalization and conspiracy belief, with only a small profile showing higher levels of violent radicalization. A slightly larger profile was expected to show higher levels of conspiracy belief. Following the identification of distinct profiles based on these factors, the aim was to understand these profiles better by exploring how they differed in terms of gender, age, education, migration background, financial strain, extreme political orientation, and psychological distress.

## Methods

### Participants

Data for this cross-sectional study were collected through an online survey as part of a larger project on violent radicalization and conspiracy belief among adolescents and emerging adults. For this study, measures of violent radicalization, conspiracy belief, and individual characteristics were used. Dutch-speaking youth between 16 and 25 years old were eligible to participate. Participants were recruited using a variety of methods to achieve a diverse sample of Dutch youth. The questionnaire was distributed among students of secondary and post-secondary education institutions (18 institutions; *n* = 1800). It was also distributed on the social media platforms of Reddit, Facebook, YouTube, Instagram, and TikTok (*n* = 316) and flyers and posters were distributed at political events, train stations, and protests (*n* = 83). A small subset of participants reportedly found the survey via their social network (*n* = 93). To minimize duplicate participation across contexts, Qualtrics’ ‘prevent ballot box stuffing’ feature was used. In addition, the final dataset was reviewed for duplicate email addresses. The current study was part of a larger data collection effort in which participants received a €5.00 gift card for completing the first part of the study and were additionally eligible for a raffle to win a €50.00 gift card if they also completed the second part.

In total, 2474 participants within the age range of 16 to 25 years old participated in the study. Participants who did not progress beyond the first part of the questionnaire were excluded (*n* = 108), leaving 2366 participants. Participants who failed the attention check were flagged for review, as such checks can help identify inattentive respondents (Oppenheimer et al., 2009). If these participants were also outliers in terms of other indicators of inattention, specifically, inconsistent responding (e.g., Dupuis et al., 2019), completion time (e.g., Curran, 2016), or monotone responding (e.g., Meade & Craig, 2012), they were excluded (*n* = 69), leaving a final sample of 2297 adolescents and emerging adults.

Of these 2297 participants (*M*_*age*_ = 19.55, *SD*_*age*_ = 2.75), about two-thirds identified as female (63.3%; *n* = 1453), about a third as male (34.0%; *n* = 782) and smaller subgroups self-identified (1.1%; *n* = 25), or preferred not to state their gender (1.6%; *n* = 37). At the time of participation, the majority of participants were in secondary vocational education (40.4%; *n* = 928), followed by university education (26.9%; *n* = 617), and secondary education (22.0%; *n* = 506). A small subgroup worked fulltime (5.3%; *n* = 122), parttime (3.7%; *n* = 86), or was unemployed (1.7%; *n* = 38). Additionally, a small group was born abroad (i.e., first-generation immigrant; 4.8%, *n* = 111) or was born in the Netherlands but one or both parents were born abroad (i.e., second-generation immigrant; 15.2%, *n* = 348). In terms of political orientation, a large group placed themselves in the exact center of the political spectrum (26.9%; *n* = 619), with a little less than half identifying as left of center (45.3%; *n* = 1040) and about a quarter as right of center (27.8%; *n* = 638). Two small subgroups chose the most extreme left-wing option (2.5%; *n* = 57) or the most extreme right-wing option (2.0%; *n* = 47).

### Procedure

Ethical approval for this study was obtained from the Ethics Review Board of the Faculty of Social & Behavioral Sciences of Utrecht University (24-0377) and the study was preregistered on OSF (https://osf.io/v72r8/). Although the use of multiple imputation for handling missing data was preregistered, full information maximum likelihood (FIML) was used instead, given the low rate of missingness and the limited analytic flexibility for imputed data in Mplus. Participants were presented with a digital information letter and were directed to the study if they consented to participating in the study and confirmed they were at least 16 years old. At the participating educational institutions, information on the study was also briefly presented to the classes and the first author and/or a trained Master student were available to answer questions in person.

### Materials

#### Profile variables

##### Violent radical attitudes

Violent radical attitudes (VRA) were measured using the short, 8-item version (Frounfelker et al., 2021) of the Sympathies for Violent Radicalization and Terrorism Scale (SyfoR; Bhui et al., 2014). Participants were asked to “Rate your support for or condemnation of the following actions as part of political protests…” on items such as “violence to fight police injustice”, using a 5-point Likert scale ranging from 1 (*total disapproval*) to 5 (*total approval*), with higher scores indicating stronger violent radical attitudes. The scale showed good internal consistency (ω = 0.87).

##### Violent radical intentions

Violent radical intentions (VRI) were assessed using the radical subscale (RIS) of the Activism and Radical Intentions Scale (ARIS; Moskalenko & McCauley, 2009). Participants indicated how likely they were to engage in each of four behaviors (e.g., “I would attack police or security forces if I saw them beating members of my group”) on a 7-point Likert scale ranging from 1 (*very unlikely*) to 7 (*very likely*), with higher scores indicating stronger violent radical intentions. The scale showed good internal consistency (ω = 0.85).

##### Violent radical behavior towards property

Violent radical behavior towards property (VRB property) was measured using five items to assess violence towards property (De Waele & Pauwels, 2014). Participants were asked “Have you ever …” followed by five statements on violence towards property (e.g., “damaged someone’s property because of your political or religious beliefs.”). They were asked to choose one of four answer options: 0 (*never*), 1 (*once*), 2, (*two or three times*), or 3 (*more than three times*). The items were dichotomized both to be consistent with original scoring (De Waele & Pauwels, 2014) and because the low prevalence of repeated behavior in the current sample made ordinal categories too small for analysis. A sum score of 0 was coded as not having engaged in violent radical behavior (0) and any sum score above 0 was coded as having engaged in violent radical behavior (1).

##### Violent radical behavior towards people

Violent radical behavior towards people (VRB people) was measured using four items to assess violence towards people (De Waele & Pauwels, 2014). Participants were asked “Have you ever …” followed by four statements on violence towards people (e.g., “threatened someone in the streets because of their political or religious beliefs”). Items were coded using the same method as for the VRB property scale.

##### General conspiracy belief

General conspiracy belief (GCB) was assessed using the Adolescent Conspiracy Belief Questionnaire (ACBQ; Jolley et al., 2021). Participants rated nine items (e.g., “The government monitors people in secret.”) on a 7-point Likert scale ranging from 1 (*strongly disagree*) to 7 (*strongly agree*), with higher scores indicating stronger general conspiracy belief. The scale showed good internal consistency (ω = 0.93).

##### Specific conspiracy belief

Specific conspiracy belief (SCB) was assessed by asking participants to rate ten specific conspiracy theories (e.g., “5G (masts) present a threat to public health.”) on a five-point Likert scale, ranging from 1 (*strongly disagree*) to 5 (*strongly agree*) with higher scores indicating stronger conspiracy beliefs. Participants could also indicate they were *unfamiliar* with a specific theory, which was treated as missing data. The proportion of participants selecting this option ranged from 3.1% to 27.7% across items. A brief pilot study was conducted in 2023 (approved by the Ethics Review Board of the Faculty of Social & Behavioral Sciences of Utrecht University, number 22-0602) to identify what specific conspiracy theories were relevant to Dutch youth. The selection of initial theories was based on Dutch news articles and social media. A sample of 198 young adults (*M*_*age*_ = 23.80, *SD*_*age*_ = 1.79) consisting of 144 women, 44 men, and 9 non-binary people indicated their familiarity, agreement, peers’ perceived agreement, and believability of these conspiracy theories. They could also provide feedback on the phrasing and suggest additional theories they considered relevant. Based on their responses, two theories that more than 50% of youth had never heard of were excluded and two were added based on suggestions of other relevant theories. The final scale showed good internal consistency (ω = 0.92).

#### **Individual Characteristics**

Individual characteristics variables were recoded into binary variables to facilitate clear comparisons across latent profiles and minimize issues related to sparse data in certain categories.

##### Gender

Participants reported their gender (*male, female, prefer to self-identify, prefer not to say*). Due to the small number of participants (*n* = 62) who self-identified their gender or chose not to disclose their gender, their responses could not be meaningfully included in the gender-based comparisons across latent profiles. Hence, these participants were excluded from profile comparisons involving gender but were retained in all other analyses. So for analytical purposes, *gender* was recoded as binary (1 = *male*, 0 = *female*).

##### Age

Participants’ age was calculated based on the difference between their date of birth and response date.

##### Education Type

Current work or education was assessed by asking what best applied: *I am in secondary education* (followed by what type: *preparatory secondary vocational education, senior general secondary education,* or *pre-university education*), *I am in tertiary education* (followed by what type: *secondary vocational education, higher professional education,* or *university education*), *I work fulltime (32* *h per week or more), I work parttime (less than 32* *h per week)*, or *I am unemployed*. This was recoded into participants’ education type with 1 = *academic* (i.e., *senior general secondary education, pre-university education, higher professional education,* or *university education*) and 0 = *vocational* (i.e., *preparatory secondary vocational education* or *secondary vocational education*). Participants not currently enrolled in education were excluded from these comparisons due to the focus on education type (*n* = 246), but were retained in all other analyses.

##### Migration background

Migration background was measured by asking where participants and their parents were born and subsequently categorized as 1 = *migration background* (participant or at least one parent born outside the Netherlands) and 0 = *native* (participant and parents born in the Netherlands).

##### Financial strain

Financial strain was measured by asking participants how frequently their family had trouble paying bills on time. Responses were recoded into 1 = *present* (*very rarely, sometimes, often, most of the time, always*) and 0 = *absent* (*never*). This item has been previously used in a Dutch youth sample (Duindam et al., 2025).

##### Political orientation

Political orientation was assessed on a scale of 0 (*extreme left*) to 10 (*extreme right*) with 5 indicating the center with one item (i.e., “In politics, the terms “left” and “right” are often used to describe political positions. Where would you place yourself on the political left-right scale of 0–10? 0 means extreme left, 10 means extreme right and 5 means political center”). This was recoded into 1 = *extreme* (i.e., score of 0–2 or 8–10) and 0 = *moderate* (i.e., score of 3–7) in line with previous research (e.g., Wagner-Egger et al., 2022; Walter & Drochon, 2022).

##### Psychological distress

Psychological distress was examined using a brief checklist developed for this study to capture a limited number of emotional and behavioral symptoms. Item selection and phrasing was loosely inspired by the Child Behavior Checklist (CBCL; Achenbach & Rescorla, 2001), which is a more comprehensive scale intended for broader clinical use. Participants indicated whether they experienced one or more emotional and behavioral symptoms in the past six months, including low mood, anxiety, trouble sleeping, difficulties in social activities or relationships, repetitive behaviors, perceptual disturbances (e.g., hearing or seeing things others do not), trouble concentrating, excessive substance use, or eating difficulties. A sum score was created to reflect overall psychological distress, with higher scores indicating a greater number of reported symptoms.

#### Analysis

Analyses were conducted in Mplus v8.10 (Muthén & Muthén, 2023). Missing data were handled using full information maximum likelihood (FIML). First, a confirmatory factor analysis (CFA) was used to assess to what extent items of the continuous profile variables (i.e., VRA, VRI, GCB, and SCB) aligned with the hypothesized factor structure. Although most scales were previously validated, a CFA was used to assess model fit in the current sample and to evaluate the structure of the newly developed SCB scale (Brown, 2015). Factor scores were extracted to reduce measurement error and to provide more precise estimates of the underlying constructs in subsequent analyses (Brown, 2015). Model fit was evaluated using the Comparative Fit Index (CFI), Root Mean Square Error of Approximation (RMSEA), and Standardized Root Mean Square Residual (SRMR).

Next, a latent profile analysis (LPA) was conducted to identify profiles of violent radicalization among adolescents and emerging adults in the context of conspiracy belief, using the resulting factor scores and two categorical indicators (VRB property and VRB people).^1^ Models with increasing numbers of profiles were estimated and compared to determine the optimal solution. Model selection was guided by a combination of statistical fit indices and interpretability. Specifically, the Akaike Information Criterion (AIC), Bayesian Information Criterion (BIC), and sample-size adjusted BIC (adj-BIC) were used, with lower values indicating better fit (Ferguson et al., 2020). Entropy values and mean posterior class probabilities were also examined to assess classification precision, with higher values indicating better separation between profiles (Masyn, 2013). Finally, the bootstrap likelihood ratio test (BLRT) was used to assess whether adding a profile significantly improved the model fit (Nylund et al., 2007). After determining the number of profiles, differences between profiles on violent radicalization and conspiracy belief measures were assessed using Wald tests and corrections for multiple testing were applied using the Benjamini-Hochberg procedure (Benjamini & Hochberg, 1995). To avoid over-reliance on statistical significance and to identify practically meaningful differences, Cohen’s *d* was calculated for continuous variables (VRA, VRI, GCB, and SCB) and Cohen’s *h* for binary variables (VRB property and people). Differences with medium or larger effect sizes (i.e., > 0.5; Cohen, 2013) were interpreted as meaningful and are discussed in the main text.

Subsequently, the characteristics of the estimated profiles were described by exploring differences in gender, age, education type, migration background, financial strain, extreme political orientation, and psychological distress across profiles. These variables were included as auxiliary variables using the BCH method (Asparouhov & Muthen, 2021), which conducts a Wald chi-square test and allows for unbiased comparisons of class-specific means and proportions while accounting for classification uncertainty. To control for multiple testing, the Benjamini–Hochberg procedure was applied (Benjamini & Hochberg, 1995). Similar to differences across indicator variables, Cohen’s *d* was calculated for continuous variables (age and psychological distress), and Cohen’s *h* for binary variables (gender, education type, migration background, financial strain, and political orientation). Differences with medium or larger effect sizes (i.e., > 0.5; Cohen, 2013) were interpreted as meaningful and are discussed in the main text. Full significance testing results and effect sizes are reported in the supplementary materials.

Sensitivity analyses were conducted to assess the robustness of the findings. Specifically, the analyses of gender and education type differences were run using a subset of participants. To assess whether this impacted the results, all analyses were rerun with these restricted samples. Additionally, to assess the missing data handling method, analyses were rerun using a single imputed dataset instead of FIML.

## Results

### Descriptives

First, the descriptive statistics for the indicator variables in the full sample were examined. Scores of violent radical attitudes (VRA; *M* = 2.21, *SD* = 0.70) and violent radical intentions (VRI; *M* = 3.17, *SD* = 1.33) were relatively low. For VRI, a small subset of participants exceeded the cutoff of 5.00 (7.5%; *n* = 169), which the authors of the scale considered indicative of violent radical intentions (Moskalenko & McCauley, 2009). Additionally, small subsets of participants reported having previously engaged in violent radical behavior against property (7.0%; *n* = 218) or people (6.9%; *n* = 215). Overall, scores on the general conspiracy belief (GCB; *M* = 3.05, *SD* = 1.28) and specific conspiracy belief (SCB; *M* = 2.05, *SD* = 0.86) scales were also relatively low. More specifically, about a third of the sample agreed or strongly agreed with two or more specific conspiracy theories (31.6%; *n* = 725), while a small subset agreed or strongly agreed with more than half of the theories (4.1%; *n* = 94).

### Model Fit

Item-level descriptives and missing data rates are reported in the supplementary materials. Item nonresponse was low, ranging from 0% to 2.8%. A CFA was conducted to assess to what extent items of the continuous profile variables (i.e., VRA, VRI, GCB, and SCB) aligned with underlying factor structures. Initial model fit was insufficient, χ^2^ (428, *N* = 2297) = 4841.64, *p* < 0.001, CFI = 0.84, RMSEA = 0.067, SRMR = 0.059. Item 8 and 9 of GCB, item 7 and 8 of VRA, and item 1 and 2 of VRI shared the same stems (differing only in the sentence endings), which can induce common method bias if the additional associations are not represented in the model (Podsakoff et al., 2003). Therefore, their residuals were allowed to correlate. This improved the model fit, χ^2^ (425, *N* = 2297) = 3419.30, *p* < 0.001, CFI = 0.89, RMSEA = 0.055, SRMR = 0.049. The initial and final model results are reported in the supplementary materials. Factor scores were exported from the final model and used for the LPA. Correlations between these factor scores and individual characteristics are presented in Table 1.

**Table 1:** Overview of correlations between factor scores of indicator variables and individual characteristics

	N	1	2	3	4	5	6	7	8	9	10	11	12	13
1. VRA	2260	—												
2. VRI	2243	0.90***	—											
3. VRB – Property (ref: no VRB)	2238	0.27***	0.28***	—										
4. VRB – People (ref: no VRB)	2233	0.27***	0.26***	–	—									
5. GCB	2297	0.55***	0.48***	0.20***	0.20***	—								
6. SCB	2267	0.50***	0.39***	0.17***	0.18***	0.93***	—							
7. Male (ref: female)	2235	0.20***	0.20***	–	–	0.03	0.004	—						
8. Age	2297	−0.26***	−0.19***	−0.02	−0.06**	−0.21***	−0.26***	−0.02	—					
9. Academic education (ref: vocational)	2050	−0.18***	−0.13***	–	–	−0.36***	−0.41***	–	0.33***	—				
10. Migration background (ref: no migration background)	2297	0.19***	0.17***	–	–	0.26***	0.23***	–	−0.06**	–	—			
11. Financial strain (ref: no financial strain)	2297	0.09***	0.09***	–	–	0.13***	0.09***	–	0.11***	–	–	—		
12. Extreme political orientation (ref: political moderate)	2297	0.11***	0.14***	–	–	−0.02	−0.06**	–	0.15***	–	–	–	—	
13. Psychological distress	2297	0.06**	0.08***	0.09***	0.07**	0.05*	0.001	−0.14***	0.09***	−0.02	0.05*	0.26***	0.04*	—

*N* refers to the number of observed cases after data processing.

*VRA* violent radical attitudes, *VRI* violent radical intentions, *VRB* violent radical behavior, *GCB* general conspiracy belief, *SCB* specific conspiracy belief

* *p* < 0.05 ** *p* < 0.01 *** *p* < 0.001

### Profiles

Table 2 indicates the fit statistics of LPA models with one through seven profiles. The six-profile model was identified as the best model based on fit and interpretability. Scores on AIC, BIC, and adjusted BIC were lower and entropy was higher for the six-profile model than the five-profile model. Though the mean posterior class probability was lower for the six-profile model, it was high in both models. The bootstrap likelihood ratio test was significant, indicating that the six-profile model provided a better fit than the five-profile model. The seven-profile model indicated marginally better fit statistics than the six-profile model, but was conceptually less meaningful, as it primarily split one profile into two profiles, without added interpretive or theoretical value. The six-profile model was therefore preferred for its interpretability and parsimony.

**Table 2:** Model fit statistics of models with different number of profiles

Number of profiles	AIC	BIC	adj-BIC	Entropy	Smallest profile size (%)	BLRT p-value	Mean posterior class probabilities
1	23208.19	23265.59	23233.81	–	2,297 (100)	–	1.00
2	19469.24	19566.81	19512.80	0.88	791 (34.4)	<0.001	0.96
3	18166.39	18304.13	18227.88	0.91	207 (9.0)	<0.001	0.95
4	17227.47	17405.39	17306.89	0.86	342 (14.9)	<0.001	0.92
5	16554.25	16772.35	16651.62	0.87	103 (4.5)	<0.001	0.92
6	16009.50	16267.77	16124.80	0.88	84 (3.7)	<0.001	0.91
7	15642.76	15941.20	15775.99	0.87	97 (4.2)	<0.001	0.90

*AIC* Akaike Information Criterion. *BIC* Bayesian Information Criterion, *adj-BIC* Sample-size adjusted Bayesian Information Criterion, *BLRT* Bootstrap likelihood ratio test

**Fig. 1:**
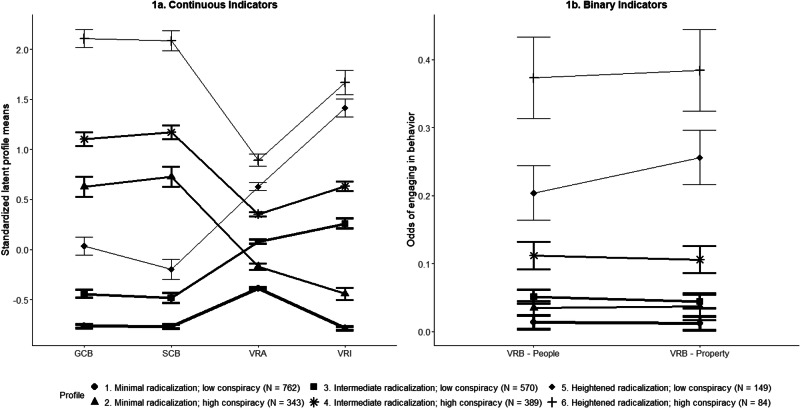
Standardized latent profile means of continuous (**1a**) and odds of binary (**1b**) profile indicators including accompanying standard errors for the six-profile model. VRA violent radical attitudes, VRI violent radical intentions, GCB general conspiracy belief, SCB specific conspiracy belief, VRB violent radical behavior. Each line represents a latent profile with the thickness of the lines indicating profile size. Values reflect standardized latent profile means (**1a**) and odds (**1b**) across the six profile indicators

The six profiles are visualized in Fig. 1 and summarized in the supplementary materials, including pairwise comparisons and effect sizes > 0.5. Profile labels were assigned based on the overall pattern of violent radicalization and conspiracy belief, as informed by medium or larger sized standardized differences (>0.5) in indicator variables across profiles. The *minimal radicalization; low conspiracy* profile (33.2%; *n* = 762), was the largest profile, with below average levels of all measures of violent radicalization and conspiracy belief, the lowest of all profiles. The *minimal radicalization; high conspiracy* profile (14.9%; *n* = 343) showed similar levels of violent radicalization as the other minimal radicalization profile, but above-average levels of conspiracy belief. The *intermediate radicalization; low conspiracy* profile (24.8%; *n* = 570) had around average levels of violent radicalization combined with below average levels of conspiracy belief. The *intermediate radicalization; high conspiracy* profile (16.9%; *n* = 389) had slightly above average levels of violent radicalization, similar to the other intermediate radicalization profile, combined with above average levels of conspiracy belief. The *heightened radicalization; low conspiracy* profile (6.5%; *n* = 149) had above average levels of violent radicalization, combined with slightly below average levels of conspiracy belief. Finally, the *heightened radicalization; high conspiracy* profile (3.7%; *n* = 84) was the smallest profile and had above average levels of violent radicalization, similar to the other heightened radicalization profile, and above average levels of conspiracy belief.

### Individual Characteristics

To further describe the profiles, differences on individual characteristics were assessed based on significance and effect sizes. Only medium or larger sized differences (>0.5) are discussed here. Table 3 presents the individual characteristics per profile as well as medium or larger sized differences (>0.5) in pairwise comparisons. Gender differed significantly across profiles, χ^2^(5, *N* = 2235) = 117.67, *p* < 0.001. The percentage of men in each profile ranged from 23.0% in the *minimal radicalization; high conspiracy* profile to 70.1% in the *heightened radicalization; high conspiracy* profile. The overall pattern indicated that the heightened radicalization profiles had a higher percentage of men than the minimal and intermediate radicalization profiles. The only exception was that no medium sized difference was found between the *heightened radicalization; low conspiracy* profile and the *intermediate radicalization; low conspiracy* profile.

**Table 3:** Percentages, means, and standard errors for individual characteristics across six profiles

Characteristics	Male gender (ref: female)	Age (*M, SE*)	Academic education (ref: vocational)	Migration background (ref: no migration background)	Financial strain (ref: absent)	Extreme political orientation (ref: moderate)	Psychological distress (*M, SE*)
1. Minimal radicalization; low conspiracy	27.7% ^5,6^	20.55 (0.11) ^2,4,5,6^	76.6% ^2,4,6^	11.5% ^4,6^	22.8%	28.6%	2.37 (0.08)
2. Minimal radicalization; high conspiracy	23.0% ^,5,6^	19.14 (0.15) ^1^	28.6% ^1,3,5^	22.9% ^6^	29.1%	17.2% ^5,6^	2.67 (0.13)
3. Intermediate radicalization; low conspiracy	42.9% ^6^	19.41 (0.14)	73.4% ^2,4,6^	13.8% ^6^	26.2%	34.0%	2.67 (0.10)
4. Intermediate radicalization; high conspiracy	32.0% ^5,6^	18.66 (0.13) ^1^	34.4% ^1,3^	34.1% ^1^	42.9%	26.0%	2.77 (0.13)
5. Heightened radicalization; low conspiracy	59.6% ^1,2.4^	18.93 (0.25) ^1^	57.8% ^2,6^	27.2%	37.3%	49.4% ^2^	3.41 (0.23) ^6^
6. Heightened radicalization; high conspiracy	70.1% ^1,2,3,4^	18.32 (0.27) ^1^	31.2% ^1,3,5^	46.9% ^1,2,3^	29.3%	48.4% ^2^	2.29 (0.27) ^5^
*Sample total*	*34.0%*	*19.55* (*0.04)*	*58.6%*	*20.0%*	*29.2%*	*29.9%*	*2.62 (0.03)*

Superscript numbers indicates profiles from which the current profile differs significantly (*p* < 0.05), based on pairwise comparisons, after applying the Benjamini-Hochberg procedure to control for multiple comparisons and with an effect size (Cohen’s *d* or *h*) of > 0.5

Mean age differed significantly across profiles, χ^2^(5, *N* = 2297) = 168.94, *p* < 0.001. The average age across profile means ranged from 18.32 years in the *heightened radicalization; high conspiracy* profile to 20.55 years in the *minimal radicalization; low conspiracy* profile. The *minimal radicalization; low conspiracy* profile was older than other profiles. The only exception was that no medium sized difference was found for the comparison to the *intermediate radicalization; low conspiracy* profile.

Education type differed significantly across profiles, χ^2^(5, *N* = 2050) = 357.40, *p* < 0.001. The percentage of the sample that was enrolled in an academic education track ranged from 28.6% in the *minimal radicalization; high conspiracy* profile to 76.6% in the *minimal radicalization; low conspiracy* profile. The overall pattern indicated low conspiracy profiles had a higher proportion of academic education than the high conspiracy profiles. The only exception was that no medium sized difference was found between the *intermediate radicalization; high conspiracy* profile and the *heightened radicalization; low conspiracy* profile.

Migration background differed significantly across profiles, χ^2^(5, *N* = 2297) = 112.67, *p* < 0.001. The percentage of participants with a migration background ranged from 11.5% in the *minimal radicalization; low conspiracy* profile to 46.9% in the *heightened radicalization; high conspiracy* profile. Overall, the *heightened radicalization; high conspiracy* profile had a higher proportion of migration background than the minimal radicalization profiles and the *intermediate radicalization; low conspiracy* profile. The *minimal radicalization; low conspiracy* profile also had a lower proportion of migration background than the *intermediate radicalization; high conspiracy* profile.

Financial strain differed significantly across profiles, χ^2^(5, *N* = 2297) = 47.12, *p* < 0.001. Experiences of financial strain ranged from 22.8% in the *minimal radicalization; low conspiracy* profile to 42.9% in the *intermediate radicalization; high conspiracy* profile but no differences across profiles were medium or larger sized.

Extreme political orientation differed significantly across profiles, χ^2^ (5, *N* = 2297) = 62.64, *p* < 0.001. The percentage of participants with a more extreme political orientation ranged from 17.2% in the *minimal radicalization; high conspiracy* profile to 49.4% in the *heightened radicalization; low conspiracy* profile. Overall, higher levels of extreme political orientation were found in the heightened radicalization profiles, compared to the *minimal radicalization; high conspiracy* profile.

Finally, psychological distress differed significantly across profiles, χ^2^ (5, *N* = 2297) = 26.86, *p* < 0.001. The average number of emotional and behavioral symptoms ranged from 2.29 in the *heightened radicalization; high conspiracy* profile to 3.41 in the *heightened radicalization; low conspiracy* profile. Psychological distress was higher in the *heightened radicalization; low conspiracy* profile than in the *heightened radicalization; high conspiracy* profile.

### Sensitivity Analyses

Sensitivity analyses showed that the overall profile structure and interpretations remained consistent when restricting the sample by gender or education status, or when using a single imputed dataset. Full results for all three sensitivity analyses are provided in the supplementary materials.

## Discussion

Violent radicalization linked to conspiracy belief has gained increasing attention (Basit, 2021), with youth being especially at risk (Enders et al., 2021; Neumann & Rogers, 2007). Therefore, the purpose of this study was to identify profiles of adolescents and emerging adults based on differences in conspiracy belief and violent radicalization. Additionally, differences across profiles were explored in terms of individual characteristics, specifically, gender, age, education type, migration background, financial strain, political orientation, and psychological distress.

Six distinct profiles were identified, varying in levels of violent radicalization and conspiracy belief, underscoring the heterogeneity of violent radicalization and conspiracy belief among adolescents and emerging adults. The first and largest profile, the *minimal radicalization; low conspiracy* profile (33.2%; *n* = 762), was characterized by below average levels of all measures of violent radicalization and conspiracy belief. The second profile, *minimal radicalization; high conspiracy* (14.9%; *n* = 343), was characterized by violent radicalization levels that were similar to those in the *minimal radicalization; low conspiracy* profile, while conspiracy belief was above average. The third, the *intermediate radicalization; low conspiracy* profile (24.8%; *n* = 570), was characterized by around average levels of violent radicalization combined with below average levels of conspiracy belief. The fourth, the *intermediate radicalization; high conspiracy* profile (16.9%; *n* = 389), was characterized by slightly above average levels of violent radicalization, combined with relatively high levels of conspiracy belief. The fifth profile, the *heightened radicalization; low conspiracy* profile (6.5%; *n* = 149) was characterized by above average levels of violent radicalization, combined with slightly below average levels of conspiracy belief. Finally, the sixth and smallest profile, the *heightened radicalization; high conspiracy* profile (3.7%; n = 84), was characterized by above average levels of violent radicalization and above average levels of conspiracy belief.

On average, levels of violent radicalization and conspiracy belief were relatively low across the sample. However, approximately 10% of youth were classified into a heightened radicalization profile and about 35% into a high conspiracy profile, suggesting that a notable subgroup of adolescents and emerging adults expressed elevated levels of violent radicalization and, even more so, conspiracy belief. These findings are in line with prior person-centered research on violent radicalization that found most youth falling into lower violent radicalization profiles, and only a smaller subset belonging to higher violent radicalization profiles (e.g., Duindam et al., 2025; Schröder et al., 2022). The current study builds on this previous research by incorporating conspiracy belief, offering a better understanding of how it interacts with violent radicalization in adolescence and emerging adulthood. For example, the identification of multiple profiles with heightened violent radicalization but differing in conspiracy belief supports earlier findings that link the two concepts (Belton et al., 2025), suggesting that conspiracy belief may be linked to violent radicalization for some individuals but not others. Notably, the identification of a small profile high in both conspiracy belief and violent radicalization aligns with concerns raised by institutions such as the European parliament (Kruglova, 2023). At the same time, the presence of larger profiles with high conspiracy belief but low violent radicalization supports the view that only a small subset of conspiracy believers might violently radicalize (Moskalenko & McCauley, 2021).

Overall, while cognitive and behavioral radicalization tended to co-occur in the heightened radicalization profiles, this was less consistent in the intermediate and minimal radicalization profiles. Across these profiles, cognitive radicalization levels (violent radical attitudes and intentions) varied, intentions even more so than attitudes, whereas violent radical behavior was generally low. This separation of cognitive and behavioral radicalization aligns with theoretical models such as the two-pyramids model (McCauley & Moskalenko, 2017), which conceptualize these two as distinct but related processes.

Furthermore, levels of general and specific conspiracy belief were relatively similar within profiles. Theoretically, greater variability was expected in specific conspiracy belief compared to general conspiracy belief, as the latter reflects a broader disposition, whereas the former targets more specific beliefs with a lower prevalence (Imhoff, Bertlich, et al., 2022). Some nuance may have been lost by using factor scores based on specific conspiracy theory items, as previous research suggests that belief in individual conspiracy theories relates differently to violent radicalization (Enders et al., 2024).

### Heightened Radicalization Profiles

The heightened radicalization profiles (one with low, and one with high conspiracy belief) both consisted of a male majority, that was relatively younger and held more extreme political opinions, compared to the minimal or intermediate radicalization profiles. These patterns are consistent with previous studies associating violent radicalization with male gender, (Moskalenko et al., 2023), younger age (Bhui et al., 2014), and extreme political orientation (Wagner-Egger et al., 2022). Financial strain was broadly similar to other profiles.

Despite similarities, these two heightened radicalization profiles differed in relative frequency of education type. The *heightened radicalization; high conspiracy* profile included 31.2% academically educated youth, whereas the *heightened radicalization; low conspiracy* profile included 57.8%. This pattern was present across other profiles as well, with high conspiracy profiles consistently including lower proportions of academically educated youth, compared to the low conspiracy belief profiles. This aligns with previous findings on conspiracy belief and education that found lower education levels were associated with increased conspiracy belief (Douglas et al., 2016). This association may be explained through higher education being associated with greater cognitive complexity, which makes individuals less likely to accept simple solutions to complex problems (Van Prooijen, 2017). Higher education is also associated with increased perceived control, which could reduce the appeal of conspiracy theories that offer seemingly coherent, but misleading, narratives (Van Prooijen, 2017).

Furthermore, youth in the *heightened radicalization; low conspiracy* profile indicated significantly more psychological distress than the *heightened radicalization; high conspiracy profile*. Psychological distress has previously been associated with violent radicalization (Adam-Troian & Bélanger, 2024; Bhui et al., 2020), especially when combined with high conspiracy belief (e.g., Baum et al., 2024; Levinsson et al., 2021). This would suggest the highest levels of psychological distress would be found in the *heightened radicalization; high conspiracy* profile. However, in our study, psychological distress was lower in this group compared to the low conspiracy counterpart. One possible explanation is that the measure of psychological distress in this study captured the number of symptoms masking nuances in symptom type or severity. This could have obscured differences between the two profiles. For example, while youth in the *heightened radicalization; low conspiracy* profile reported most symptoms more frequently than youth in the *heightened radicalization; high conspiracy* profile, the latter more often reported hallucinatory-type experiences (19.0% vs. 8.1%). The higher prevalence of hallucinatory-type experiences does align with previous research that found a high rate of paranoid schizophrenia, bipolar disorder, and Munchausen syndrome by proxy in QAnon offenders (Jensen & Kane, 2024). However, it does not align with other studies that reported the highest level of violent radicalization for those with both strong conspiracy belief and depression and anxiety (Baum et al., 2024; Levinsson et al., 2021). In the current findings, internalizing difficulties were more present in the *heightened radicalization; low conspiracy* profile. This discrepancy may partly reflect methodological differences as those prior studies used variable-centered approaches, while the current study used a person-centered approach, suggesting that the relationship between conspiracy belief, psychological distress, and violent radicalization may differ across individuals.

Migration background was relatively more prevalent in the *heightened radicalization; high conspiracy* profile. This finding align with research associating migration background with violent radicalization (Thijssen et al., 2023) and conspiracy belief (Van Prooijen et al., 2018). However, it contrasts with previous research in Canadian youth, which found lower levels of violent radicalization (Levinsson et al., 2021) and conspiracy belief (Levinsson et al., 2022) among youth with a migration background. One explanation may lie in contextual differences between Canada and the Netherlands in terms of immigration. It could be that immigration policies and public attitudes in the Netherlands tend to be more restrictive and assimilation-oriented (Entzinger, 2014) compared to Canada’s less restrictive, more multicultural approach (Paquet & Lawlor, 2022). This, in turn, may shape how youth with a migration background experience factors such as belonging, identity, and exclusion, which could more easily trigger a need for significance in the Dutch sample, making them more vulnerable to violent radicalization and conspiracy belief (Kruglanski et al., 2022).

Taken together, both heightened radicalization profiles seem to reflect relatively small groups of youth characterized by above average levels of violent radicalization and individual characteristic previously linked to violent radicalization, namely male gender, younger age, and extreme political orientation. These profiles could be considered higher-risk compared to other profiles, due to their elevated violent radical intentions and behavior. Despite similarities of these profiles, differences in individual characteristics might play a role in the different levels of conspiracy belief found in these heightened radicalization profiles. Specifically, the *heightened radicalization; high conspiracy* profile represents a group of more vocationally educated youth, more often with a migration background. In contrast, the *heightened radicalization; low conspiracy* profile reflects a more academically educated subgroup experiencing stronger psychological distress. These findings underscore the possibility of multiple pathways to violent radicalization, shaped by varying social, psychological, and cognitive influences.

### Intermediate Radicalization Profiles

The intermediate radicalization profiles (one with low, and one with high conspiracy belief) were composed of a majority of women and politically moderate youth. These youth were somewhat younger than those in the minimal radicalization profiles and migration background and psychological distress were broadly in line with other profiles. As with the heightened radicalization profiles, the high conspiracy profile included more vocationally educated youth, while the low conspiracy profile consisted of more academically educated youth.

Financial strain was highest in the *intermediate radicalization; high conspiracy* profile, but differences across profiles did not reach a medium effect size (i.e., Cohen’s *d* or *h* > 0.5), although previous studies found that financial strain was more likely in more violently radicalized individuals (e.g., Thijs et al., 2024) as well as those with higher conspiracy belief (e.g., Walter & Drochon, 2022). One explanation for the limited variation in our sample is that the Netherlands is characterized by relatively lower levels of income inequality (Hasell et al., 2023) and strong social support systems (Di Carlo et al., 2025), which may have reduced the relevance of financial strain as a distinguishing factor. Another possible explanation is that the measure of financial strain specifically asked how often families had difficulty paying bills on time, which may not fully capture broader or more subjective experiences of financial strain. For instance, some adolescent participants may have be unaware of financial issues in their family, or emerging adult participants may have more personal financial issues, separate from the familial context.

Taken together, these profiles seem to reflect a more intermediate subgroup with higher violent radical attitudes and intentions than the minimal radicalization profiles but smaller differences in terms of violent radical behavior. As in other profile pairs, the two profiles differed primarily in their levels of conspiracy belief.

### Minimal Radicalization Profiles

The minimal radicalization profiles (one with low, and one with high conspiracy belief) were both characterized by older, predominantly female samples. Differences in education type showed similar patterns as in the heightened and intermediate radicalization profiles. Financial strain, migration background, and psychological distress aligned broadly with other profiles and extreme political orientation was less common than in the intermediate and heightened radicalization profiles.

The low proportion of men in the *minimal radicalization; high conspiracy* profile aligns with findings suggesting stronger conspiracy belief among women (Moskalenko et al., 2023), but contrasts with studies showing higher belief among men (Schuster et al., 2023). One possible explanation may be the moderating role of gender in the association between conspiracy belief and violent radicalization. Prior research indicated a stronger link between conspiracy belief and violent radical intentions in men than in women (Moskalenko et al., 2023). These findings align with the high proportion of men in the *heightened radicalization; high conspiracy* profile and may help explain the higher proportion of women in the *minimal radicalization; high conspiracy* profile. Together, these findings suggest that conspiracy belief may co-occur with violent radicalization more often in men than in women.

Interestingly, the *minimal radicalization; high conspiracy* profile included a lower proportion of extreme political orientations than the *minimal radicalization; low conspiracy* profile, despite previous research finding higher prevalence of conspiracy belief at the extremes of the political spectrum (Imhoff, Zimmer, et al., 2022; Van Prooijen et al., 2015). However, previous research also found that people with stronger conspiracy belief tend to feel more disconnected from or skeptical about the effectiveness of political systems (Ardèvol-Abreu et al., 2020). A substantial share of youth in the *minimal radicalization; high conspiracy* profile also selected the exact center of the political spectrum (38.8%), possibly reflecting political disengagement or ambivalence and suggesting that conspiracy belief in this profile may be reflected in a more general mistrust of political systems rather than extreme political ideologies. Relatedly, conspiracy belief might serve as a generalized political attitude (Imhoff & Bruder, 2014). From the perspective of the 3N model (Kruglanski et al., 2022), both conspiracy belief and extreme political orientation might function as ‘narratives’ that offer explanations for perceived grievances. It is therefore possible that, for some youth, one narrative may reduce the psychological need for the other (Kruglanski et al., 2014). However, others have argued that conspiracy beliefs may represent a distinct worldview rather than a generalized political attitude (Sutton & Douglas, 2020). In general, it is important to consider the developmental stage of participants. Political orientation continues to develop during adolescence and emerging adulthood (Dinas, 2013) and is still shaped by influences such as parents (Cooperman, 2023; Van Ditmars, 2023). The measure itself also assumes a certain level of political knowledge and engagement, so the political center response may also reflect uncertainty or unfamiliarity with terms like ‘left’ and ‘right’. Overall, the differences in political orientation across profiles highlight the complexity of political orientation in adolescents and emerging adults through the ongoing development of political identity and understanding.

Taken together, both minimal radicalization profiles seem to reflect larger, lower-risk groups of relatively older, politically moderate, and predominantly female youth with below average levels of violent radicalization. Both minimal radicalization profiles reflect youth who are least likely to support or engage in violent radical behavior. However, they differ in conspiracy belief, with the high conspiracy profile also characterized by political moderation (or, perhaps, ambivalence), aligning with the perspective that conspiracy belief does not always co-occur with violent radicalization (Moskalenko & McCauley, 2021).

### Strengths and Limitations

The findings of this study should be interpreted in light of its limitations. The first limitation relates to causal inference and interpretation of the findings. Specifically, while experimental evidence suggests conspiracy belief could increase violent radical intentions (Imhoff et al., 2021), the cross-sectional design does not allow for causal conclusions so the current findings cannot determine the direction of this relationship. Additionally, the identified profiles represent a valuable snapshot in time for understanding current patterns of vulnerability among youth, but they may shift as societal conditions or intervention efforts evolve. Finally, while individual differences across profiles are valuable in contextualizing the profiles, demographic patterns such as education type are likely explained by underlying psychological factors (Hornsey et al., 2023) and indicative of broader societal context. For example, differences in education type may be explained by differences in feelings of powerlessness, which has been associated with both lower educational attainment and stronger conspiracy belief (Van Prooijen, 2017).

Furthermore, there is a potential risk of social desirability and nonresponse bias, particularly given the sensitive nature of the study topic. This could lead to underreporting of violent radicalization and conspiracy belief as participants may be concerned for repercussions or misrepresentation. To mitigate these risks, minimal (optional) personal information was requested and data handling procedures were clearly explained. Moreover, the heterogeneity found in both conspiracy belief and violent radicalization suggests that participants were generally willing to report varying levels of these concepts, despite the sensitivity of the topic.

Finally, the sample had a gender imbalance, with approximately two-thirds of the sample identifying as women and one-third as men. This may have influenced the findings, particularly given the gender differences observed across profiles. For example, profiles with a male majority (i.e., those characterized by heightened violent radicalization) may have appeared more extreme in contrast to the overall female-skewed sample, where average levels of violent radicalization were lower. It is also possible that these male-majority profiles would have been larger or more differentiated in a more gender-balanced sample. Nonetheless, the overall sample included a substantial number of men (*n* = 782), supporting the reliability of gender-based comparisons.

Notwithstanding these limitations, this study expands on previous research by using a person-centered approach to identify distinct profiles of adolescents and emerging adults based on their levels of violent radicalization and conspiracy belief, which offers a more detailed overview of how these factors manifest in middle to late adolescence and emerging adulthood. While person-centered approaches have previously been used to study violent radicalization, this study builds on that work by incorporating conspiracy belief as an additional indicator. The current study also includes self-report behavioral measures of violent radicalization, which have been limited thus far (Belton et al., 2025), allowing for a more comprehensive understanding of violent radicalization (McCauley & Moskalenko, 2017). Finally, by focusing specifically on adolescents and emerging adults, the study addresses a vulnerable population in this field (Duindam et al., 2025; Emmelkamp et al., 2020), offering insights into how these constructs manifest during a formative developmental period.

### Future Research

The current study identified distinct profiles based on violent radicalization and conspiracy belief, suggesting multiple pathways toward violent radicalization. A key next step is to examine how risk and protective factors are associated with profile membership and to further explore these potential pathways. Such analyses should not only focus on heightened radicalization profiles but also investigate the factors that protect individuals in lower-risk profiles, providing insight into potential resilience to violent radicalization and conspiracy belief, which can inform prevention efforts.

More longitudinal and experimental research is also needed to understand developmental processes and gain insight into causal mechanisms. For example, some profiles might violently radicalize due to belief in conspiracy theories, while others may be exposed to such theories after becoming more violently radicalized. Longitudinal studies can also provide valuable insights into how these profiles evolve over time, for example, whether individuals in different profiles radicalize or deradicalize, and how the role of conspiracy belief may vary between profiles.

Finally, this study aimed to provide a broad overview of these profiles by including multiple brief measures of individual differences. However, future research could benefit from more comprehensive and validated measures of variables such as financial strain, psychological distress, and political orientation. Additionally, examining belief in specific conspiracy theories instead of relying on a combined score, could reveal unique contributions to different profiles.

### Implications

The identification of six distinct profiles of youth with varying levels of violent radicalization and conspiracy belief as well as differences in individual characteristics, highlights the importance of differentiated prevention strategies that are grounded in social-developmental models (Beelmann, 2020) and public health frameworks (Eisenman & Flavahan, 2017). At the individual level, more intensive and targeted interventions may be appropriate for the heightened radicalization profiles (Andrews et al., 2011). Given limited robust evidence for interventions that directly reduce violent radicalization (Jugl et al., 2020), it may be useful to draw from youth violence prevention more generally, where a public health approach has already been implemented (Eisenman & Flavahan, 2017). For addressing stronger conspiracy belief, interpersonal approaches show promise (Hornsey et al., 2023) and may be even more relevant for adolescents, considering their sensitivity to the opinions of peers (Capella et al., 2023). Broader prevention programs that promote skills such as critical thinking, and civic engagement were also found to be effective (Brouillette-Alarie et al., 2022) and could be widely implemented in schools. These broader prevention programs may not only reduce the appeal of violence but also promote more general goals of positive youth development (Law & Atkinson, 2021). A recent example of this is the introduction of mandatory civic education in Dutch vocational schools, which would focus on democratic processes, misinformation, and personal development (Ministerie van Onderwijs Cultuur en Wetenschap, 2024). However, in line with socio-ecological models, prevention should extend beyond the individual level to also address institutional and societal factors that create fertile ground for violent radicalization and conspiracy belief. These processes emerge not only from individual factors but also from a broader context of legitimate grievances, mistrust, and institutional failure (Hornsey et al., 2023; Miconi et al., 2024), which in turn can trigger a need for significance and fuel both conspiracy belief and violent radicalization (Kruglanski et al., 2022). For example, youth with migration backgrounds and those reporting higher psychological distress were more prevalent in the heightened radicalization profiles, suggesting that these groups may be especially affected by unmet needs and systemic inequalities. A recent illustration of these systemic challenges in the Dutch context is the major childcare benefits scandal, where thousands of families (disproportionately from migrant backgrounds) were wrongly accused of fraud through discriminatory profiling (Tweede Kamer der Staten-Generaal, 2020). Addressing these structural injustices is therefore not only a matter of equality, but also appears to be a key component of effective prevention.

## Conclusion

Violent radicalization in the context of conspiracy belief can have large-scale societal consequences. However, little research has been done on this specific topic in youth. Therefore, the purpose of this study was to identify profiles of adolescents and emerging adults, aged 16–25, based on differences in conspiracy belief and violent radicalization and explore differences across profiles in terms of individual characteristics. Six heterogeneous profiles of adolescents and emerging adults with varying levels of violent radicalization and conspiracy belief were identified. While the largest profile indicated low levels of both, two smaller profiles indicated heightened violent radicalization, with differing levels of conspiracy belief. Generally, more violently radicalized profiles were younger, male, and more politically extreme whereas participants in profiles with stronger conspiracy beliefs were more frequently vocationally educated. Differences in migration background and psychological distress were also observed across profiles. These patterns reflect not just individual differences but also broader societal contexts. This perspective is consistent with socio-ecological and public health approaches, which view violent radicalization as the result of interactions between individual and environmental factors. Overall, these findings underscore that violent radicalization is limited to a small subset of adolescents and emerging adults, while conspiracy belief is more prevalent among this group. Although they can co-occur, these findings illustrate the diversity in how conspiracy belief and violent radicalization manifest, and highlight the importance of considering individual and broader contextual conditions.

## Supplementary information

Supplementary Materials

## Footnotes

1 An attempt was made to run a factor mixture model combining the CFA and LPA, but this was numerically unstable, which is why the analysis was simplified by splitting it into two steps.
