# Supplementary material for: Understanding Violent Radicalization and Conspiracy Belief in Dutch Youth Aged 16–25: A Latent Profile Analysis
Source: J Youth Adolesc. 2025 Sep 17;55(2):269–88. doi: 10.1007/s10964-025-02250-4 (PMC12894200; doi:10.1007/s10964-025-02250-4)
Supplement: Supplementary file 1 — Supplementary Materials [file 10964_2025_2250_MOESM1_ESM.pdf]

Supplementary Materials

Title: Understanding Violent Radicalization and Conspiracy Belief in Dutch Youth Aged 16-25: A Latent Profile Analysis

Authors: Jessica I. den Elzen, Jessica J. Asscher , Kyle M. Lang , Hanne M. Duindam

Standardized latent profile means, odds, and standard errors for violent radicalization and conspiracy belief across six profiles.

Profile	VRA	VRI	VRB - Property	VRB - People	GCB	SCB	N (%)
	M (SE)	*M (SE)*	*Odds of engaging in behavior (SE)*	*Odds of engaging in behavior (SE)*	*M (SE)*	*M (SE)*	
1. Minimal radicalization; low conspiracy	-0.39 (0.01)[4,5,6]	-0.79 (0.02)[3,4,5,6]	0.004 (0.01) [5,6]	0.01 (0.01) [5,6]	-0.77 (0.02)[2,4,5,6]	-0.77 (0.02) [2,4,5,6]	762 (33.2)
2. Minimal radicalization; high conspiracy	-0.17 (0.03)[4,5,6]	-0.44 (0.06)[3,4,5,6]	0.04 (0.02) [5,6]	0.04 (0.01) [5,6]	0.63 (0.10)[1,3,5,6]	0.73 (0.10)[1,3,5,6]	343 (14.9)
3. Intermediate radicalization; low conspiracy	0.08 (0.02)[5,6]	0.26 (0.05)[1,2,5,6]	0.04 (0.01) [5,6]	0.05 (0.01) [6]	-0.45 (0.04)[2,4,5,6]	-0.49 (0.05)[2,4,6]	570 (24.8)
4. Intermediate radicalization; high conspiracy	0.35 (0.02)[1,2,6]	0.63 (0.05)[1,2,5,6]	0.11 (0.02) [6]	0.11 (0.02) [6]	1.10 (0.07)[1,3,5,6]	1.17 (0.07)[1,3,5,6]	389 (16.9)
5. Heightened radicalization; low conspiracy	0.63 (0.04)[1,2,3]	1.41 (0.09)[1,2,3,4]	0.26 (0.04) [1,2,3]	0.20 (0.04) [1,2]	0.04 (0.09)[1,2,3,4,6]	-0.20 (0.10)[1,2,4,6]	149 (6.5)
6. Heightened radicalization; high conspiracy	0.89 (0.06)[1,2,3,4]	1.67 (0.12)[1,2,3,4]	0.38 (0.06) [1,2,3,4]	0.37 (0.06) [1,2,3,4]	2.11 (0.09)[1,2,3,4,5]	2.08 (0.10)[1,2,3,4,5]	84 (3.7)

Note. N = 2,297. VRA = violent radical attitudes, VRI = violent radical intentions, VRB = violent radical behavior, GCB = general conspiracy belief, SCB = specific conspiracy belief. Superscript numbers indicates profiles from which the current profile differs significantly ($p < .05$), based on pairwise comparisons, after applying the Benjamini-Hochberg procedure to control for multiple comparisons and with an effect size (Cohen's d or h) of > 0.5.

Item-level descriptives and missing data rates.

Item	M	SD	N
ATT_1: Commit minor crime.	2.87	1.03	2,260
ATT_2: Use violence.	1.66	0.95	2,260
ATT_3: Organise radical groups without personally taking part.	1.70	0.93	2,260
ATT_4: Use bombs to fight injustice.	1.39	0.86	2,260
ATT_5: Violence to protect family.	3.22	1.18	2,260
ATT_6: Violence by organized groups to protect own race/religious group.	2.19	1.09	2,260
ATT_7: Violence to fight police injustice.	2.32	1.21	2,260
ATT_8: Violence to fight government injustice.	2.32	1.2	2,260
RIS_1: I would continue to support an organization that fights for my group's political and legal rights even if the organization sometimes breaks the law.	4.02	1.61	2,243
RIS_2: I would continue to support an organization that fights for my group's political and legal rights even if the organization sometimes resorts to violence.	3.16	1.62	2,243
RIS_3: I would participate in a public protest against oppression of my group even if I thought the protest might turn violent.	2.88	1.76	2,243

RIS_4: I would attack police or security forces if I saw them beating members of my group.	2.62	1.71	2,243
CT_1: The truth about the harmful side effects of vaccinations is deliberately hidden from the general public.	2.35	1.30	2,189
CT_2: All major media are secretly colluding with the government.	2.38	1.21	2,171
CT_3: The negative effects of climate change are deliberately exaggerated by scientists.	2.23	1.26	2,192
CT_4: 5G (masts) pose a danger to public health.	1.87	1.05	2,083
CT_5: Nitrogen emissions from farmers do not contribute nearly as much to climate problems as the government and scientists claim.	2.68	1.38	2,149
CT_6: The World Economic Forum (WEF) is pulling the strings in the West and driving society toward The Great Reset.	2.06	1.13	1,645
CT_7: The world is ruled by reptiles posing as humans with powerful positions.	1.47	0.96	2,112
CT_8: The number of Jews murdered by the Nazis during World War II is deliberately exaggerated.	1.52	0.89	2,169
CT_9: The COVID-19 coronavirus has been intentionally used by governments to attain power.	1.88	1.16	2,205
CT_10: The attacks on September 11, 2001, were staged by the U.S. government.	1.75	1.09	2,111
ACBQ_1: The government deliberately hides important information from the public.	4.13	1.67	2,297
ACBQ_2: The government monitors people in secret.	3.74	1.74	2,297
ACBQ_3: Some political groups have secret plans which are not good for society.	4.05	1.76	2,297
ACBQ_4: Some diseases have been created by the government to be used as weapons.	2.3	1.68	2,297

ACBQ_5: The government often knows about terrorist attacks and lets them happen.	2.36	1.57	2,297
ACBQ_6: Governments have deliberately spread diseases in certain groups of people	2.22	1.59	2,297
ACBQ_7: Secret groups control people’s minds without them knowing.	2.36	1.66	2,297
ACBQ_8: Secret societies control politicians and other leaders.	2.96	1.79	2,297
ACBQ_9: Secret societies influence many political decisions.	3.33	1.83	2,297

Note: Items from the specific conspiracy belief (CT) scale were originally developed in Dutch and have been translated into English for interpretability. For all other scales, the original English item phrasing is presented.

Initial CFA Model

MODEL:

```
VRA  BY att_1 att_2 att_3 att_4 att_5 att_6 att_7 att_8;
VRI  BY ris_1 ris_2 ris_3 ris_4;
GCB  BY acbq_1 acbq_2 acbq_3 acbq_4 acbq_5 acbq_6 acbq_7 acbq_8 acbq_9;
SCB  BY ct_1 ct_2 ct_3 ct_4 ct_5 ct_6 ct_7 ct_8 ct_9 ct_10;
```

MODEL RESULTS

	Estimate	S.E.	Est./S.E.	Two-Tailed P-Value
VRA BY				
ATT_1	1.000	0.000	999.000	999.000
ATT_2	1.200	0.073	16.500	0.000
ATT_3	0.953	0.064	14.963	0.000
ATT_4	0.808	0.070	11.503	0.000
ATT_5	1.582	0.102	15.473	0.000
ATT_6	1.695	0.106	16.022	0.000
ATT_7	2.377	0.167	14.230	0.000
ATT_8	2.479	0.170	14.615	0.000
VRI BY				
RIS_1	1.000	0.000	999.000	999.000
RIS_2	1.188	0.028	42.213	0.000
RIS_3	1.059	0.043	24.837	0.000

RIS_4	0.944	0.046	20.582	0.000

GCB BY

ACBQ_1	1.000	0.000	999.000	999.000
ACBQ_2	1.019	0.030	33.665	0.000
ACBQ_3	0.923	0.034	26.815	0.000
ACBQ_4	1.374	0.052	26.386	0.000
ACBQ_5	1.204	0.045	26.730	0.000
ACBQ_6	1.281	0.049	26.235	0.000
ACBQ_7	1.251	0.047	26.450	0.000
ACBQ_8	1.504	0.048	31.465	0.000
ACBQ_9	1.478	0.046	32.171	0.000

SCB BY

CT_1	1.000	0.000	999.000	999.000
CT_2	0.883	0.021	42.492	0.000
CT_3	0.821	0.023	35.086	0.000
CT_4	0.640	0.023	27.734	0.000
CT_5	0.849	0.025	34.594	0.000
CT_6	0.864	0.023	37.192	0.000
CT_7	0.538	0.027	19.734	0.000
CT_8	0.523	0.025	20.937	0.000
CT_9	0.928	0.022	42.238	0.000
CT_10	0.723	0.026	27.305	0.000

VRI WITH

VRA	0.346	0.030	11.569	0.000

GCB WITH

VRA	0.178	0.018	10.120	0.000
VRI	0.439	0.033	13.148	0.000

SCB WITH

VRA	0.160	0.016	9.760	0.000
VRI	0.367	0.031	11.829	0.000
GCB	0.871	0.038	23.147	0.000

Intercepts

ACBQ_1	4.132	0.035	118.406	0.000
ACBQ_2	3.744	0.036	103.355	0.000
ACBQ_3	4.052	0.037	110.385	0.000
ACBQ_4	2.296	0.035	65.473	0.000
ACBQ_5	2.364	0.033	72.101	0.000
ACBQ_6	2.216	0.033	66.944	0.000
ACBQ_7	2.363	0.035	68.391	0.000
ACBQ_8	2.958	0.037	79.339	0.000
ACBQ_9	3.327	0.038	87.287	0.000
CT_1	2.375	0.028	86.264	0.000
CT_2	2.402	0.026	93.617	0.000
CT_3	2.246	0.027	83.689	0.000
CT_4	1.900	0.023	82.249	0.000
CT_5	2.702	0.029	91.824	0.000

CT_6	2.105	0.025	83.225	0.000
CT_7	1.503	0.022	69.647	0.000
CT_8	1.540	0.020	78.161	0.000
CT_9	1.907	0.025	76.887	0.000
CT_10	1.792	0.024	74.525	0.000
ATT_1	2.869	0.022	132.844	0.000
ATT_2	1.662	0.020	82.714	0.000
ATT_3	1.702	0.019	87.400	0.000
ATT_4	1.388	0.018	76.951	0.000
ATT_5	3.223	0.025	129.506	0.000
ATT_6	2.195	0.023	95.768	0.000
ATT_7	2.319	0.025	91.241	0.000
ATT_8	2.320	0.025	91.909	0.000
RIS_1	4.021	0.034	118.705	0.000
RIS_2	3.168	0.034	92.591	0.000
RIS_3	2.885	0.037	77.791	0.000
RIS_4	2.619	0.036	72.782	0.000

Variances

VRA	0.177	0.021	8.220	0.000
VRI	1.292	0.072	17.927	0.000
GCB	0.967	0.061	15.916	0.000
SCB	1.054	0.041	26.018	0.000

Residual Variances

ACBQ_1	1.831	0.051	36.131	0.000

ACBQ_2	2.011	0.061	32.990	0.000
ACBQ_3	2.272	0.061	37.264	0.000
ACBQ_4	1.000	0.051	19.417	0.000
ACBQ_5	1.067	0.052	20.506	0.000
ACBQ_6	0.932	0.054	17.290	0.000
ACBQ_7	1.228	0.051	23.948	0.000
ACBQ_8	1.005	0.047	21.280	0.000
ACBQ_9	1.225	0.054	22.881	0.000
CT_1	0.639	0.027	23.929	0.000
CT_2	0.645	0.026	24.954	0.000
CT_3	0.881	0.033	26.342	0.000
CT_4	0.679	0.027	25.478	0.000
CT_5	1.148	0.039	29.177	0.000
CT_6	0.445	0.024	18.364	0.000
CT_7	0.619	0.033	18.670	0.000
CT_8	0.518	0.025	20.558	0.000
CT_9	0.447	0.022	20.308	0.000
CT_10	0.644	0.030	21.307	0.000
ATT_1	0.877	0.027	32.192	0.000
ATT_2	0.656	0.031	21.356	0.000
ATT_3	0.695	0.024	29.030	0.000
ATT_4	0.618	0.031	19.949	0.000
ATT_5	0.960	0.033	29.179	0.000
ATT_6	0.680	0.029	23.811	0.000
ATT_7	0.462	0.047	9.839	0.000
ATT_8	0.357	0.039	9.215	0.000

RIS_1	1.289	0.053	24.218	0.000
RIS_2	0.811	0.057	14.331	0.000
RIS_3	1.640	0.076	21.684	0.000
RIS_4	1.759	0.074	23.909	0.000

Final CFA Model

MODEL:

```
VRA     BY att_1 att_2 att_3 att_4 att_5 att_6 att_7 att_8;
VRI     BY ris_1 ris_2 ris_3 ris_4;
GCB     BY acbq_1 acbq_2 acbq_3 acbq_4 acbq_5 acbq_6 acbq_7 acbq_8 acbq_9;
SCB     BY ct_1 ct_2 ct_3 ct_4 ct_5 ct_6 ct_7 ct_8 ct_9 ct_10;
acbq_8  WITH acbq_9;
att_7   WITH att_8;
ris_1   WITH ris_2;
```

MODEL RESULTS

	Estimate	S.E.	Est./S.E.	Two-Tailed P-Value
VRA BY				
ATT_1	1.000	0.000	999.000	999.000
ATT_2	1.310	0.075	17.543	0.000
ATT_3	1.029	0.065	15.907	0.000
ATT_4	0.853	0.073	11.710	0.000
ATT_5	1.555	0.102	15.294	0.000
ATT_6	1.666	0.106	15.719	0.000
ATT_7	1.743	0.101	17.334	0.000
ATT_8	1.901	0.110	17.264	0.000

VRI BY

RIS_1	1.000	0.000	999.000	999.000
RIS_2	1.274	0.040	32.239	0.000
RIS_3	1.330	0.053	24.863	0.000
RIS_4	1.231	0.059	21.030	0.000

GCB BY

ACBQ_1	1.000	0.000	999.000	999.000
ACBQ_2	1.016	0.030	33.365	0.000
ACBQ_3	0.916	0.035	26.346	0.000
ACBQ_4	1.442	0.054	26.469	0.000
ACBQ_5	1.249	0.047	26.589	0.000
ACBQ_6	1.335	0.051	26.124	0.000
ACBQ_7	1.266	0.049	26.003	0.000
ACBQ_8	1.435	0.047	30.745	0.000
ACBQ_9	1.390	0.044	31.916	0.000

SCB BY

CT_1	1.000	0.000	999.000	999.000
CT_2	0.879	0.021	42.367	0.000
CT_3	0.820	0.023	34.979	0.000
CT_4	0.641	0.023	27.614	0.000
CT_5	0.846	0.025	34.527	0.000
CT_6	0.862	0.023	37.066	0.000
CT_7	0.543	0.027	19.794	0.000
CT_8	0.526	0.025	20.933	0.000
CT_9	0.933	0.022	42.250	0.000

CT_10	0.725	0.027	27.364	0.000

VRI WITH

VRA	0.349	0.029	12.114	0.000

GCB WITH

VRA	0.215	0.018	12.034	0.000
VRI	0.388	0.031	12.512	0.000

SCB WITH

VRA	0.206	0.016	12.796	0.000
VRI	0.329	0.029	11.503	0.000
GCB	0.870	0.038	22.772	0.000

ACBQ_8 WITH

ACBQ_9	0.828	0.045	18.603	0.000

ATT_7 WITH

ATT_8	0.486	0.028	17.090	0.000

RIS_1 WITH

RIS_2	0.542	0.050	10.832	0.000

Intercepts

ACBQ_1	4.132	0.035	118.406	0.000
ACBQ_2	3.744	0.036	103.355	0.000

ACBQ_3	4.052	0.037	110.385	0.000
ACBQ_4	2.296	0.035	65.473	0.000
ACBQ_5	2.364	0.033	72.101	0.000
ACBQ_6	2.216	0.033	66.944	0.000
ACBQ_7	2.363	0.035	68.390	0.000
ACBQ_8	2.958	0.037	79.339	0.000
ACBQ_9	3.327	0.038	87.287	0.000
CT_1	2.376	0.028	86.276	0.000
CT_2	2.403	0.026	93.606	0.000
CT_3	2.246	0.027	83.706	0.000
CT_4	1.901	0.023	82.257	0.000
CT_5	2.702	0.029	91.822	0.000
CT_6	2.104	0.025	83.194	0.000
CT_7	1.504	0.022	69.660	0.000
CT_8	1.541	0.020	78.149	0.000
CT_9	1.908	0.025	76.918	0.000
CT_10	1.792	0.024	74.554	0.000
ATT_1	2.869	0.022	132.844	0.000
ATT_2	1.663	0.020	82.721	0.000
ATT_3	1.702	0.019	87.395	0.000
ATT_4	1.389	0.018	76.924	0.000
ATT_5	3.224	0.025	129.530	0.000
ATT_6	2.196	0.023	95.784	0.000
ATT_7	2.318	0.025	91.232	0.000
ATT_8	2.320	0.025	91.901	0.000
RIS_1	4.021	0.034	118.685	0.000

RIS_2	3.167	0.034	92.578	0.000
RIS_3	2.885	0.037	77.767	0.000
RIS_4	2.619	0.036	72.764	0.000

Variances

VRA	0.207	0.023	9.149	0.000
VRI	0.916	0.066	13.962	0.000
GCB	0.936	0.061	15.388	0.000
SCB	1.054	0.041	25.955	0.000

Residual Variances

ACBQ_1	1.861	0.051	36.298	0.000
ACBQ_2	2.047	0.061	33.338	0.000
ACBQ_3	2.309	0.061	38.012	0.000
ACBQ_4	0.879	0.047	18.588	0.000
ACBQ_5	1.009	0.051	19.960	0.000
ACBQ_6	0.849	0.052	16.368	0.000
ACBQ_7	1.241	0.053	23.468	0.000
ACBQ_8	1.266	0.048	26.296	0.000
ACBQ_9	1.527	0.052	29.424	0.000
CT_1	0.641	0.027	24.083	0.000
CT_2	0.653	0.026	25.106	0.000
CT_3	0.884	0.034	26.314	0.000
CT_4	0.679	0.027	25.410	0.000
CT_5	1.154	0.039	29.342	0.000
CT_6	0.450	0.024	18.452	0.000

CT_7	0.614	0.033	18.602	0.000
CT_8	0.516	0.025	20.483	0.000
CT_9	0.440	0.022	19.937	0.000
CT_10	0.641	0.030	21.185	0.000
ATT_1	0.847	0.027	31.314	0.000
ATT_2	0.556	0.027	20.515	0.000
ATT_3	0.637	0.022	28.829	0.000
ATT_4	0.582	0.029	20.405	0.000
ATT_5	0.902	0.033	27.492	0.000
ATT_6	0.614	0.026	23.579	0.000
ATT_7	0.832	0.035	23.750	0.000
ATT_8	0.695	0.030	23.038	0.000
RIS_1	1.665	0.057	29.063	0.000
RIS_2	1.148	0.061	18.684	0.000
RIS_3	1.472	0.078	18.906	0.000
RIS_4	1.523	0.070	21.743	0.000

Sensitivity Analysis Gender

Analyses without participants who self-identified or preferred not to state their gender. The final CFA model had similar fit as in the original dataset, X^2 (425, N = 2,235) = 3264.46, p < .001, CFI = .90, RMSEA = .055, SRMR = .048.

Table 1. Model fit statistics of models with different number of profiles for data excluding participants who self-identified or preferred not to state their gender.

Number of profiles	AIC	BIC	adj-BIC	Entropy	Smallest profile size (%)	BLRT p-value	Mean posterior class probabilities
1	22306.422	22363.542	22331.770	-	2235 (100%)	-	1.00
2	18608.328	18705.432	18651.421	.88	771 (34.5)	< .001	.96
3	17336.083	17473.171	17396.919	.91	205 (9.2)	< .001	.95
4	16448.659	16625.731	16527.239	.86	345 (15.4)	< .001	.92
5	15790.648	16007.703	15886.971	.87	102 (4.6)	< .001	.92
6	15303.912	15560.952	15417.979	.87	85 (3.8)	< .001	.91
7	14899.486	15196.510	15031.298	.86	96 (4.3)	< .001	.89

Table 2. Standardized latent profile means, odds, and standard errors for violent radicalization and conspiracy belief across six profiles for data excluding participants who self-identified or preferred not to state their gender.

Profile	VRA	VRI	VRB - Property	VRB - People	GCB	SCB	N (%)
	M (SE)	*M (SE)*	*Odds of engaging in behavior (SE)*	*Odds of engaging in behavior (SE)*	*M (SE)*	*M (SE)*	
1. Minimal radicalization; low conspiracy	-0.37 (0.01)	-0.78 (0.03)	0.01 (0.01)	0.01 (0.01)	-0.78 (0.02)	-0.78 (0.03)	736 (32.9)
2. Minimal radicalization; high conspiracy	-0.16 (0.03)	-0.43 (0.08)	0.03 (0.02)	0.03 (0.02)	0.60 (0.14)	0.70 (0.14)	339 (15.2)

3. Intermediate radicalization; low conspiracy	0.08 (0.02)	0.26 (0.06)	0.04 (0.01)	0.05 (0.01)	-0.46 (0.06)	-0.50 (0.07)	550 (24.6)
4. Intermediate radicalization; high conspiracy	0.33 (0.03)	0.61 (0.06)	0.10 (0.02)	0.10 (0.02)	1.13 (0.09)	1.19 (0.09)	378 (16.9)
5. Heightened radicalization; low conspiracy	0.59 (0.04)	1.37 (0.11)	0.24 (0.04)	0.20 (0.04)	0.09 (0.12)	-0.12 (0.14)	147 (6.6)
6. Heightened radicalization; high conspiracy	0.84 (0.06)	1.63 (0.12)	0.39 (0.06)	0.38 (0.06)	2.11 (0.10)	2.10 (0.11)	85 (3.8)

Table 3. Percentages, means, and standard errors for individual characteristics across six profiles for data excluding participants who self-identified or preferred not to state their gender.

Characteristics	Male gender (ref: female)	Age (*M, SE*)	Academic education (ref: vocational)	Migration background (ref: no migration background)	Financial strain (ref: absent)	Extreme political orientation (ref: moderate)	Psychological distress (*M, SE*)
1. Minimal radicalization; low conspiracy	27.7%	20.59 (0.11)	76.4%	11.6%	23.2%	28.7%	2.35 (0.08)
2. Minimal radicalization; high conspiracy	23.0%	19.19 (0.15)	29.7%	20.2%	27.8%	16.7%	2.68 (0.14)

3. Intermediate radicalization; low conspiracy	42.8%	19.38 (0.14)	74.6%	13.0%	26.0%	31.8%	2.64 (0.10)
4. Intermediate radicalization; high conspiracy	31.5%	18.62 (0.13)	33.5%	34.1%	42.9%	23.1%	2.70 (0.13)
5. Heightened radicalization; low conspiracy	59.0%	18.81 (0.24)	53.6%	28.4%	36.3%	47.3%	3.37 (0.22)
6. Heightened radicalization; high conspiracy	67.5%	18.37 (0.28)	30.7%	47.8%	32.5%	49.4%	2.36 (0.27)

Sensitivity Analysis Student

Analyses without participants who indicated they worked fulltime, parttime or were unemployed. The final CFA model had similar fit as in the original dataset, X^2 (425, N = 2,050) = 2986.31, $p < .001$, CFI = .90, RMSEA = .054, SRMR = .049.

Table 1. Model fit statistics of models with different number of profiles for data excluding participants who self-identified or preferred not to state their gender.

Number of profiles	AIC	BIC	adj-BIC	Entropy	Smallest profile size (%)	BLRT p-value	Mean posterior class probabilities
1	20360.030	20416.286	20384.515	-	2,050 (100)	-	1.00
2	17039.691	17135.326	17081.316	.88	731 (35.6)	< .001	.96
3	15926.514	16061.528	15985.278	.91	175 (8.5)	< .001	.95
4	15097.344	15271.738	15173.248	.86	297 (14.5)	< .001	.92
5	14532.400	14746.173	14625.444	.87	84 (4.1)	< .001	.91
6	14014.761	14267.912	14124.944	.89	73 (3.6)	< .001	.91
7	13724.603	14017.134	13851.926	.87	79 (3.9)	< .001	.90

Table 2. **S**tandardized latent profile means, odds, and standard errors for violent radicalization and conspiracy belief across six profiles for data excluding participants who self-identified or preferred not to state their gender.

Profile	VRA	VRI	VRB - Property	VRB - People	GCB	SCB	N (%)
	M (SE)	*M (SE)*	*Odds of engaging in behavior (SE)*	*Odds of engaging in behavior (SE)*	*M (SE)*	*M (SE)*	
1. Minimal radicalization; low conspiracy	-0.38 (0.01)	-0.77 (0.03)	0.01 (0.01)	0.01 (0.01)	-0.77 (0.02)	-0.76 (0.02)	672 (32.8)
2. Minimal radicalization; high conspiracy	-0.17 (0.03)	-0.44 (0.06)	0.04 (0.02)	0.02 (0.01)	0.65 (0.09)	0.74 (0.09)	307 (15.0)

3. Intermediate radicalization; low conspiracy	0.08 (0.02)	0.26 (0.05)	0.03 (0.01)	0.05 (0.01)	-0.45 (0.04)	-0.48 (0.04)	516 (25.2)
4. Intermediate radicalization; high conspiracy	0.34 (0.02)	0.61 (0.05)	0.09 (0.02)	0.10 (0.02)	1.09 (0.07)	1.14 (0.07)	351 (17.1)
5. Heightened radicalization; low conspiracy	0.62 (0.04)	1.41 (0.10)	0.26 (0.04)	0.23 (0.04)	0.06 (0.10)	-0.18 (0.10)	131 (6.4)
6. Heightened radicalization; high conspiracy	0.88 (0.06)	1.66 (0.12)	0.36 (0.06)	0.36 (0.06)	2.10 (0.10)	2.05 (0.11)	73 (3.6)

Table 3. Percentages, means, and standard errors for individual characteristics across six profiles for data excluding participants who self-identified or preferred not to state their gender.

Characteristics	Male gender (ref: female)	Age (*M, SE*)	Academic education (ref: vocational)	Migration background (ref: no migration background)	Financial strain (ref: absent)	Extreme political orientation (ref: moderate)	Psychological distress (*M, SE*)
1. Minimal radicalization; low conspiracy	27.6%	20.23 (0.11)	76.2%	10.9%	21.8%	29.0%	2.29 (0.08)
2. Minimal radicalization; high conspiracy	20.4%	18.80 (0.14)	29.6%	23.7%	27.5%	17.3%	2.65 (0.14)

3. Intermediate radicalization; low conspiracy	41.0%	19.09 (0.13)	73.8%	13.7%	24.5%	31.9%	2.68 (0.10)
4. Intermediate radicalization; high conspiracy	30.5%	18.38 (0.13)	34.3%	31.9%	41.5%	25.3%	2.72 (0.13)
5. Heightened radicalization; low conspiracy	58.5%	18.56 (0.23)	57.7%	28.8%	39.6%	50.1%	3.56 (0.24)
6. Heightened radicalization; high conspiracy	67.2%	18.07 (0.25)	31.3%	45.8%	26.8%	48.9%	2.51 (0.31)

Sensitivity Analysis FIML

Analyses without participants who indicated they worked fulltime, parttime or were unemployed. The final CFA model had similar fit as in the original dataset, X^2 (425, N = 2,297) = 3594.02, $p < .001$, CFI = .89, RMSEA = .057, SRMR = .049.

Table 1. Model fit statistics of models with different number of profiles for data excluding participants who self-identified or preferred not to state their gender.

Number of profiles	AIC	BIC	adj-BIC	Entropy	Smallest profile size (%)	BLRT p-value	Mean posterior class probabilities
1	23428.068	23485.462	23453.690	-	2,297 (100)	-	1.00
2	19742.717	19840.286	19786.274	.88	766 (33.3)	< .001	.95
3	18467.511	18605.256	18529.003	.91	208 (9.1)	< .001	.95
4	17471.877	17649.797	17551.305	.86	354 (15.4)	< .001	.92
5	16810.385	17028.481	16907.748	.87	101 (4.4)	< .001	.92
6	16245.832	16504.103	16361.130	.88	85 (3.7)	< .001	.91
7	15875.974	16174.420	16009.207	.88	78 (3.4)	< .001	.91

Table 2. **S**tandardized latent profile means, odds, and standard errors for violent radicalization and conspiracy belief across six profiles for data excluding participants who self-identified or preferred not to state their gender.

Profile	VRA	VRI	VRB - Property	VRB - People	GCB	SCB	N (%)
	M (SE)	*M (SE)*	*Odds of engaging in behavior (SE)*	*Odds of engaging in behavior (SE)*	*M (SE)*	*M (SE)*	
1. Minimal radicalization; low conspiracy	-0.39 (0.01)	-0.79 (0.02)	0.02 (0.01)	0.01 (0.01)	-0.76 (0.02)	-0.76 (0.02)	779 (33.9)
2. Minimal radicalization; high conspiracy	-0.17 (0.02)	-0.45 (0.05)	0.04 (0.01)	0.04 (0.01)	0.63 (0.09)	0.72 (0.08)	343 (14.9)

3. Intermediate radicalization; low conspiracy	0.08 (0.02)	0.28 (0.05)	0.05 (0.01)	0.05 (0.01)	-0.45 (0.04)	-0.48 (0.04)	556 (24.2)
4. Intermediate radicalization; high conspiracy	0.37 (0.02)	0.65 (0.05)	0.11 (0.02)	0.12 (0.02)	1.10 (0.07)	1.17 (0.07)	389 (16.9)
5. Heightened radicalization; low conspiracy	0.64 (0.05)	1.44 (0.10)	0.26 (0.04)	0.20 (0.04)	0.01 (0.10)	-0.23 (0.11)	145 (6.3)
6. Heightened radicalization; high conspiracy	0.93 (0.09)	1.71 (0.16)	0.40 (0.06)	0.39 (0.06)	2.09 (0.12)	2.07 (0.14)	85 (3.7)

Table 3. Percentages, means, and standard errors for individual characteristics across six profiles for data excluding participants who self-identified or preferred not to state their gender.

Characteristics	Male gender (ref: female)	Age (*M, SE*)	Academic education (ref: vocational)	Migration background (ref: no migration background)	Financial strain (ref: absent)	Extreme political orientation (ref: moderate)	Psychological distress (*M, SE*)
1. Minimal radicalization; low conspiracy	27.1%	20.58 (0.11)	75.9%	11.5%	23.3%	28.8%	2.37 (0.08)
2. Minimal radicalization; high conspiracy	21.9%	19.12 (0.15)	33.1%	22.2%	29.2%	15.7%	2.68 (0.13)

3. Intermediate radicalization; low conspiracy	44.4%	19.32 (0.14)	74.3%	13.7%	25.6%	34.0%	2.68 (0.10)
4. Intermediate radicalization; high conspiracy	32.0%	18.66 (0.13)	35.8%	35.7%	42.1%	26.9%	2.75 (0.13)
5. Heightened radicalization; low conspiracy	58.2%	19.15 (0.26)	58.8%	25.7%	39.3%	51.5%	3.45 (0.23)
6. Heightened radicalization; high conspiracy	71.4%	18.25 (0.27)	30.1%	46.6%	29.9%	48.1%	2.25 (0.28)

Individual Characteristics Significance Testing and Effect Sizes

Table 1. Percentages, means, and standard errors for individual characteristics across six profiles with pairwise significance testing.

Characteristics	Male gender (ref: female)	Age (*M, SE*)	Academic education (ref: vocational)	Migration background (ref: no migration background)	Financial strain (ref: absent)	Extreme political orientation (ref: moderate)	Psychological distress (*M, SE*)
1. Minimal radicalization; low conspiracy	27.7% [3,5,6]	20.55 (0.11) [2,3,4,5,6]	76.6% [2,4,5,6]	11.5% [2,4,5,6]	22.8% [4,5]	28.6% [2,5,6]	2.37 (0.08) [3,4,5]
2. Minimal radicalization; high conspiracy	23.0% [3,4,5,6]	19.14 (0.15) [1,4,6]	28.6% [1,3,5]	22.9% [1,3,4,6]	29.1% [4]	17.2% [1,3,4,5,6]	2.67 (0.13) [5]
3. Intermediate radicalization; low conspiracy	42.9% [1,2,4,5,6]	19.41 (0.14) [1,4,6]	73.4% [2,4,5,6]	13.8% [2,4,5,6]	26.2% [4,5]	34.0% [2,4,5,6]	2.67 (0.10) [1,5]
4. Intermediate radicalization; high conspiracy	32.0% [2,3,5,6]	18.66 (0.13) [1,2,3]	34.4% [1,3,5]	34.1% [1,2,3]	42.9% [1,2,3,6]	26.0% [2,3.5,6]	2.77 (0.13) [1,5]
5. Heightened radicalization; low conspiracy	59.6% [1,2.3,4]	18.93 (0.25) [1]	57.8% [1,2,3,4,6]	27.2% [1,3,6]	37.3% [1,3]	49.4% [1,2,3,4]	3.41 (0.23) [1,2,3,4,6]
6. Heightened radicalization; high conspiracy	70.1% [1,2,3,4]	18.32 (0.27) [1,2,3]	31.2% [1,3,5]	46.9% [1,2,3,5]	29.3% [4]	48.4% [1,2,3,4]	2.29 (0.27) [5]

Note. Superscript numbers indicates profiles from which the current profile differs significantly ($p < .05$), based on pairwise comparisons, after applying the Benjamini-Hochberg procedure to control for multiple comparisons.

Table 2. Effect sizes for significant differences across groups.

Variable	Profile	Profile	Difference	ES
Male gender (ref: female)	1	3	15.2	.320
		5	31.9	.655
		6	42.4	.876
	2	3	19.9	.428
		4	9.0	.202
		5	36.6	.764
		6	47.1	.984
	3	4	10.9	.226
		5	16.7	.336
		6	27.2	.556
	4	5	27.6	.561
		6	38.1	.782
Age	1	2	1.41	.512
		3	1.14	.414
		4	1.89	.686
		5	1.62	.588
		6	2.23	.809
	2	4	.48	.174
		6	.82	.298
	3	4	.75	.272
		6	1.09	.396
Academic education (ref: vocational)	1	2	48.0	1.003

		4	42.2	.878
		5	18.8	.404
		6	45.4	.946
	2	3	44.8	.929
		5	29.2	.599
	3	4	39.0	.804
		5	15.6	.330
		6	42.2	.873
	4	5	23.4	.474
	5	6	26.6	.542
Migration background (ref: no migration background)	1	2	11.4	.306
		4	22.6	.555
		5	15.7	.405
		6	35.4	.817
	2	3	9.1	.237
		4	11.2	.249
		6	24.0	.511
	3	4	20.3	.486
		5	13.4	.336
		6	33.1	.748
	5	6	19.7	.411
Financial strain (ref: absent)	1	4	20.1	.433
		5	14.5	.318
	2	4	13.8	.289

	3	4	16.7	.354
		5	11.1	.239
	4	6	13.6	.284
Extreme political orientation (ref: moderate)	1	2	11.4	.273
		5	20.8	.430
		6	19.8	.410
	2	3	16.8	.390
		4	8.8	.215
		5	32.3	.704
		6	31.2	.684
	3	4	8.0	.175
		5	15.4	.314
		6	14.4	.294
	4	5	23.4	.489
		6	22.4	.469
Psychological distress	1	3	0.30	.142
		4	0.40	.189
		5	1.04	.491
	2	5	0.74	.349
	3	5	0.74	.349
	4	5	0.64	.302
	5	6	1.12	.529
